# Supplementary material for: From sequence to enzyme mechanism using multi-label machine learning
Source: BMC Bioinformatics. 2014 May 19;15:150. doi: 10.1186/1471-2105-15-150 (PMC4229970; doi:10.1186/1471-2105-15-150)
Supplement: Additional file 2 — Java code of ml2db. Additional file ml2db_code.tar.gz contains the Java source code to run the multi-label machine learning experiments and save the results to database. The code’s Javadoc is included. [file 1471-2105-15-150-S2.zip › additional file 2/ml2db/ml2db/doc/main/ml2db/mulan/mulanarff/class-use/IdInstance.html]

Uses of Class main.ml2db.mulan.mulanarff.IdInstance


JavaScript is disabled on your browser.


- Overview
- Package
- Class
- Use
- Tree
- Deprecated
- Index
- Help

- Prev
- Next

- Frames
- No Frames

- All Classes

## Uses of Class main.ml2db.mulan.mulanarff.IdInstance

- Packages that use IdInstance

  | Package | Description |
  | --- | --- |
  |  |  |
  | --- | --- |
  | main.ml2db.al.utility |  |
  | main.ml2db.al.utility.confidence |  |
  | main.ml2db.al.utility.globaldistance |  |
  | main.ml2db.al.utility.hybrid |  |
  | main.ml2db.al.utility.neighbours |  |
  | main.ml2db.al.utility.pickers |  |
  | main.ml2db.al.utility.pickers.byfrequency |  |
  | main.ml2db.graphviz |  |
  | main.ml2db.mulan.mulanarff |  |
  | main.ml2db.mulan.predict |  |
  | test.ml2db.al |  |
  | test.ml2db.al.utility.globaldistance |  |
- - ### Uses of IdInstance in main.ml2db.al.utility

    Methods in main.ml2db.al.utility with parameters of type IdInstance

    | Modifier and Type | Method and Description |
    | --- | --- |
    |  |  |
    | --- | --- |
    | `abstract double` | UtilityMetric.`getUtility(mulan.classifier.MultiLabelLearner trainedLearner, IdInstance instance)` |
    | `abstract void` | UtilityMetric.`refresh(IdMultiLabelInstances labelled, IdMultiLabelInstances unlabelled, IdInstance queryInstance)` Refresh the set of labelled and unlabelled instances |
  - ### Uses of IdInstance in main.ml2db.al.utility.confidence

    Methods in main.ml2db.al.utility.confidence with parameters of type IdInstance

    | Modifier and Type | Method and Description |
    | --- | --- |
    |  |  |
    | --- | --- |
    | `static double[]` | ConfidenceCalculator.`getConfidences(mulan.classifier.MultiLabelLearner trainedLearner, IdInstance instance)` // input: trained model // actions: get MultiLabelOutput from trained model using // .makePredictions(Instance), // get confidence[] --> one confidence for each class label |
    | `double` | ConfidenceCalculator.`getUncertainty(mulan.classifier.MultiLabelLearner trainedLearner, IdInstance instance)` For each confidences set (double[]), calculate the uncertainty and store it. |
    | `double` | ConfidenceMetric.`getUtility(mulan.classifier.MultiLabelLearner trainedLearner, IdInstance instance)` Gets a measure of how distant the instance is from other unlabelled instances (good if it's close to them) and labelled instances (bad if it's close to them) and multiplies it by the average confidence certainty (see Confidence class). |
    | `void` | ConfidenceMetric.`refresh(IdMultiLabelInstances labelled, IdMultiLabelInstances unlabelled, IdInstance queryInstance)` |
  - ### Uses of IdInstance in main.ml2db.al.utility.globaldistance

    Methods in main.ml2db.al.utility.globaldistance with parameters of type IdInstance

    | Modifier and Type | Method and Description |
    | --- | --- |
    |  |  |
    | --- | --- |
    | `double` | GlobalDistanceMetric.`getUtility(mulan.classifier.MultiLabelLearner trainedLearner, IdInstance instance)` Gets a measure of how distant the instance is from other unlabelled instances (good if it's close to them) and labelled instances (bad if it's close to them). |
    | `void` | GlobalDistanceMetric.`refresh(IdMultiLabelInstances labelled, IdMultiLabelInstances unlabelled, IdInstance instanceLabelled)` |
  - ### Uses of IdInstance in main.ml2db.al.utility.hybrid

    Methods in main.ml2db.al.utility.hybrid with parameters of type IdInstance

    | Modifier and Type | Method and Description |
    | --- | --- |
    |  |  |
    | --- | --- |
    | `double` | GlobalDistanceWithConfidenceMetric.`getUtility(mulan.classifier.MultiLabelLearner trainedLearner, IdInstance instance)` Gets a measure of how distant the instance is from other unlabelled instances (good if it's close to them) and labelled instances (bad if it's close to them) and multiplies it by the average confidence certainty (see Confidence class). |
    | `void` | GlobalDistanceWithConfidenceMetric.`refresh(IdMultiLabelInstances labelled, IdMultiLabelInstances unlabelled, IdInstance instanceLabelled)` |
  - ### Uses of IdInstance in main.ml2db.al.utility.neighbours

    Subclasses of IdInstance in main.ml2db.al.utility.neighbours

    | Modifier and Type | Class and Description |
    | --- | --- |
    |  |  |
    | --- | --- |
    | `class` | `Neighbourhood` Contains information about: an instance, how much it is needed in this data set (the instances it is the nearest neighbour of), and how much it is labelled (whether it or its nearest or further neighbours are already labelled or partially known). |

    Methods in main.ml2db.al.utility.neighbours with parameters of type IdInstance

    | Modifier and Type | Method and Description |
    | --- | --- |
    |  |  |
    | --- | --- |
    | `double` | NeighbourhoodNetwork.`getUtility(mulan.classifier.MultiLabelLearner trainedLearner, IdInstance instance)` Only works with identified instances. |
    | `void` | NeighbourhoodNetwork.`refresh(IdMultiLabelInstances labelled, IdMultiLabelInstances unlabelled, IdInstance instanceLabelled)` |
  - ### Uses of IdInstance in main.ml2db.al.utility.pickers

    Methods in main.ml2db.al.utility.pickers that return IdInstance

    | Modifier and Type | Method and Description |
    | --- | --- |
    |  |  |
    | --- | --- |
    | `IdInstance` | RandomPicker.`getQuery(IdMultiLabelInstances unlabelled)` |

    Methods in main.ml2db.al.utility.pickers that return types with arguments of type IdInstance

    | Modifier and Type | Method and Description |
    | --- | --- |
    |  |  |
    | --- | --- |
    | `java.util.Vector<IdInstance>` | RandomPicker.`getQueries()` |

    Methods in main.ml2db.al.utility.pickers with parameters of type IdInstance

    | Modifier and Type | Method and Description |
    | --- | --- |
    |  |  |
    | --- | --- |
    | `double` | RandomPicker.`getUtility(mulan.classifier.MultiLabelLearner trainedLearner, IdInstance instance)` |
    | `void` | RandomPicker.`refresh(IdMultiLabelInstances labelled, IdMultiLabelInstances unlabelled, IdInstance queryInstance)` |
  - ### Uses of IdInstance in main.ml2db.al.utility.pickers.byfrequency

    Methods in main.ml2db.al.utility.pickers.byfrequency with parameters of type IdInstance

    | Modifier and Type | Method and Description |
    | --- | --- |
    |  |  |
    | --- | --- |
    | `void` | NearestNeighboursByFreqUtility.`refresh(IdMultiLabelInstances labelled, IdMultiLabelInstances unlabelled, IdInstance queryInstance)` |
  - ### Uses of IdInstance in main.ml2db.graphviz

    Methods in main.ml2db.graphviz with parameters of type IdInstance

    | Modifier and Type | Method and Description |
    | --- | --- |
    |  |  |
    | --- | --- |
    | `static java.lang.String` | GraphvizInstance.`getDirectedGraphvizString(IdInstance instance)` |
    | `static java.lang.String` | GraphvizInstance.`getUndirectedGraphvizString(IdInstance instance)` |

    Method parameters in main.ml2db.graphviz with type arguments of type IdInstance

    | Modifier and Type | Method and Description |
    | --- | --- |
    |  |  |
    | --- | --- |
    | `void` | GraphVizArffColoured.`colourPredictedInstances(java.util.TreeMap<IdInstance,java.lang.Double> instanceToPercentageMap)` |
  - ### Uses of IdInstance in main.ml2db.mulan.mulanarff

    Methods in main.ml2db.mulan.mulanarff that return IdInstance

    | Modifier and Type | Method and Description |
    | --- | --- |
    |  |  |
    | --- | --- |
    | `IdInstance` | IdMultiLabelInstances.`get(int index)` For test purposes only! slow! |
    | `IdInstance` | IdMultiLabelInstances.`getIdInstance(java.lang.String identifier)` |

    Methods in main.ml2db.mulan.mulanarff that return types with arguments of type IdInstance

    | Modifier and Type | Method and Description |
    | --- | --- |
    |  |  |
    | --- | --- |
    | `java.util.TreeMap<java.lang.String,IdInstance>` | IdMultiLabelInstances.`getId2IdInstanceMap()` |
    | `java.util.Set<IdInstance>` | IdMultiLabelInstances.`getIdInstance(weka.core.Instance wekaInstance)` |
    | `java.util.Vector<IdInstance>` | IdMultiLabelInstances.`getIdInstancesByAttributesString(java.lang.String instanceAttributesString)` |
    | `java.util.TreeMap<IdInstance,boolean[]>` | IdMultiLabelInstances.`getInst2LabelsPartitionMap()` |
    | `uk.ac.standrews.utils.main.maputils.OneToManyMap<IdInstance,IdInstance>` | NeighboursPrinter.`getInstanceAndNeighboursMap()` |
    | `uk.ac.standrews.utils.main.maputils.OneToManyMap<IdInstance,IdInstance>` | NeighboursPrinter.`getInstanceAndNeighboursMap()` |
    | `java.util.Set<IdInstance>` | IdMultiLabelInstances.`getToBePredicted()` |
    | `java.util.Vector<IdInstance>` | IdMultiLabelInstances.`getVector()` |
    | `uk.ac.standrews.utils.main.maputils.OneToManyMap<java.lang.String,IdInstance>` | IdMultiLabelInstances.`getWekaInst2IdInstMap()` |

    Methods in main.ml2db.mulan.mulanarff with parameters of type IdInstance

    | Modifier and Type | Method and Description |
    | --- | --- |
    |  |  |
    | --- | --- |
    | `boolean` | IdMultiLabelInstances.`addInstance(IdInstance instance)` |
    | `int` | IdInstance.`compareTo(IdInstance otherInstance)` Order by instance identifier string |
    | `boolean` | IdInstance.`equals(IdInstance otherInstance)` |
    | `boolean[]` | IdMultiLabelInstances.`generatePartition(IdInstance instance, int[] labelIndices, int numberOfLabels)` The boolean[] partition of the labels for this instance (true if the label is present, false if absent, for each of the existing labels). |
    | `java.lang.String` | IdMultiLabelInstances.`getAttributesIndexString(IdInstance instance)` |
    | `java.lang.String` | IdMultiLabelInstances.`getAttributesNamesString(IdInstance instance)` |
    | `java.lang.String` | IdMultiLabelInstances.`getLabelsString(IdInstance inst)` Get a separated list of the labels assigned to this instance. |
    | `boolean[]` | IdMultiLabelInstances.`getTrueLabels(IdInstance idInstance)` |

    Method parameters in main.ml2db.mulan.mulanarff with type arguments of type IdInstance

    | Modifier and Type | Method and Description |
    | --- | --- |
    |  |  |
    | --- | --- |
    | `static java.lang.String` | IdMultiLabelInstances.`getInst2PartitionMapString(java.util.TreeMap<IdInstance,boolean[]> inst2PartitionMap)` |
  - ### Uses of IdInstance in main.ml2db.mulan.predict

    Methods in main.ml2db.mulan.predict that return IdInstance

    | Modifier and Type | Method and Description |
    | --- | --- |
    |  |  |
    | --- | --- |
    | `IdInstance` | PredictionsSaver.`fromWekaToIdInstance(weka.core.Instance wekaInst)` Get an identified instance corresponding to this weka instance (same attributes) |

    Methods in main.ml2db.mulan.predict with parameters of type IdInstance

    | Modifier and Type | Method and Description |
    | --- | --- |
    |  |  |
    | --- | --- |
    | `int[]` | PredictionsSaver.`generatePredictions(mulan.classifier.MultiLabelOutput multiLabelOutput, IdInstance instance, IdMultiLabelInstances baseData, boolean[] trueLabels)` |
  - ### Uses of IdInstance in test.ml2db.al

    Methods in test.ml2db.al that return IdInstance

    | Modifier and Type | Method and Description |
    | --- | --- |
    |  |  |
    | --- | --- |
    | `static IdInstance` | ActiveLearningTest.`getNextStepQueryInstance(ActiveLearning activator, int step)` |
  - ### Uses of IdInstance in test.ml2db.al.utility.globaldistance

    Methods in test.ml2db.al.utility.globaldistance with parameters of type IdInstance

    | Modifier and Type | Method and Description |
    | --- | --- |
    |  |  |
    | --- | --- |
    | `void` | GlobalDistanceMetricTest.`printInst(GlobalDistanceMetric position, IdInstance i)` |

- Overview
- Package
- Class
- Use
- Tree
- Deprecated
- Index
- Help

- Prev
- Next

- Frames
- No Frames

- All Classes
